# Diagnostic Performance of the Thrombolysis in Myocardial Infarction (TIMI) Risk Score in Predicting Angiographic Triple Vessel Disease Among Patients With Non-ST-Segment Elevation Myocardial Infarction

**DOI:** 10.7759/cureus.103159

**Published:** 2026-02-07

**Authors:** Ayesha Khaqan, Aqeel Asghar, Somia Pervaiz, Zunaira Javed, Sahrish Javiad, Muhammad Zafrullah, Shabbar H Changazi

**Affiliations:** 1 Cardiology, King Fahad Medical City, Riyadh, SAU; 2 Cardiology, Punjab Institute of Cardiology, Lahore, Lahore, PAK; 3 Cardiology, Mayo Hospital Lahore, Lahore, PAK; 4 General Surgery, Provincial Headquarter Teaching Hospital Gilgit, Gilgit, PAK

**Keywords:** angiography, diagnostic performance, nstemi, timi score, triple vessel disease

## Abstract

Background

Patients with non-ST-segment elevation myocardial infarction (NSTEMI) often have three vessel disease (TVD). The Thrombolysis in Myocardial Infarction (TIMI) risk score is a commonly used bedside tool for risk stratification; however, its diagnostic accuracy for predicting TVD remains uncertain. Therefore, this study was conducted to assess the diagnostic performance of the TIMI score in the prediction of TVD among patients with NSTEMI with coronary angiography as the reference standard.

Material and methods

A total of 185 patients with NSTEMI were included in this cross-sectional study. TIMI risk scores were assessed on admission, and the patients underwent coronary angiography. Diagnostic performance was then evaluated using sensitivity, specificity, positive predictive value (PPV), and negative predictive value (NPV).

Results

The mean age of the cohort was 62.78±9.29 years, and 70.3% were male patients. TVD was angiographically validated in 85.4% of patients. Using a TIMI score > 4, the sensitivity was 71.5%, specificity 66.7%, PPV 92.6%, and NPV 28.6%. Post hoc analysis revealed significantly higher TIMI scores in patients with TVD (p<0.001), male patients (p=0.032), diabetics (p=0.041), and urban residents (p=0.028).

Conclusion

The TIMI score demonstrates moderate diagnostic performance for predicting TVD in patients with NSTEMI, with high PPV but low NPV. It could be a useful preliminary screening method; however, it cannot substitute angiography for a final, definite diagnosis.

## Introduction

Cardiovascular disease is still the leading cause of mortality in the world, creating a significant economic burden for health systems worldwide [1]. Among the continuum of acute coronary syndromes, non-ST-segment elevation myocardial infarction (NSTEMI) is highly prevalent and represents 70% of all acute myocardial infarction (AMI) cases. Among this population, the presence of multivessel coronary artery disease (CAD) is frequently seen (up to 70% prevalence in NSTEMI) [2,3]. Triple vessel disease (TVD) is defined by significant stenosis in all three major epicardial vessels. This entity is known to have an extremely grim prognosis and requires expeditious and aggressive treatment for a better chance of survival [4].

Early identification of high-risk patients for extensive coronary artery disease is very important to optimize the best therapeutic strategies. The Thrombolysis in Myocardial Infarction (TIMI) risk score is a clinical tool that was originally developed for the prognostic stratification of unstable angina/NSTEMI. This scoring system estimates the risk of adverse cardiac events in a 14-day period, based on clinical symptoms and signs, electrocardiographic findings, and cardio-specific blood markers. Despite the usefulness of the TIMI risk score for predicting clinical outcome, its performance for predicting aspects such as angiographic findings, particularly TVD, has been inconsistent between studies [5]. Its level of reliability in identifying patients with significant coronary involvement varies, and its role continues to be refined through ongoing research [6,7].

Prior studies have shown significant heterogeneity in the diagnostic accuracy of the TIMI score for predicting TVD, with reported sensitivities ranging from 53% to 75.7% and specificities between 47.9% and 83% [8,9]. These differences suggest that estimation from the TIMI risk score is not uniform across populations and clinical settings. Moreover, studies in South Asian populations are rare, and such people often possess distinct risk profiles and disease patterns that may affect the presentation or progression of coronary artery disease (CAD). These disparities emphasize the importance of conducting specific studies to determine the validity and reliability of the TIMI risk score among these patients in the South Asian population [10]. Therefore, the objective of the current study was to evaluate the diagnostic performance of the TIMI risk score in predicting TVD in patients with NSTEMI. Also, exploratory post hoc analyses were performed to assess the relationship of TIMI scores with several demographic and clinical variables, which will provide further insight into factors that could influence the utility of this risk assessment tool in this patient population.

## Materials and methods

This cross-sectional study was conducted at the Department of Cardiology, Shaikh Zayed Hospital, Lahore, Pakistan, from April 2020 to October 2025. Ethical approval was obtained from the Institutional Review Board of Shaikh Khalifa Bin Zayed Al-Nahyan Medical and Dental College, Lahore (approval number: 03/IRB/2020). The sample size of 185 was calculated using the WHO sample size calculator for diagnostic studies, based on an expected sensitivity of 53% from previous literature [8], a 10% margin of error, 95% confidence level, and anticipated TVD prevalence of 70% in patients with NSTEMI. Patients aged between 40 and 80 years presenting with NSTEMI admitted via the emergency department were enrolled in the study using consecutive sampling. NSTEMI was defined based on the presence of ischemic symptoms, elevated troponin I levels greater than 0.01 ng/mL/ 99th percentile upper reference limit (URL), and ST-segment depressions of less than 1 mm observed on electrocardiogram (ECG). Exclusion criteria comprised individuals with valvular heart disease, a history of prior coronary artery bypass grafting, recurrent myocardial infarction, and those who had been on statin therapy for more than one year. These exclusions were applied to minimize confounding from prior revascularization, structural heart disease, previous myocardial injury, and the potential plaque-modifying effects of long-term statin therapy, thereby allowing a clearer assessment of the TIMI risk score’s diagnostic performance in treatment-naïve, native-vessel CAD. All participants provided informed consent prior to inclusion in the study.

Demographic and clinical characteristics were prospectively recorded for each patient at the time of inclusion. Demographic data, including age, sex, and body mass index (BMI) of the patient, were documented. Residential status was also recorded (urban or rural). Clinical data, including smoking history, family history of myocardial infarction, and hypertension or diabetes, were also noted. Low-density lipoprotein (LDL) cholesterol levels were also measured by laboratory analysis on all subjects.

The TIMI risk score for unstable angina/NSTEMI was calculated for each patient upon admission. The TIMI risk score, as originally published by Antman et al. [5], is a validated prognostic tool that assigns points for seven variables: age ≥ 65 years, ≥3 traditional risk factors for coronary artery disease, known coronary stenosis ≥ 50%, ST-segment deviation on admission ECG, ≥2 anginal events in the prior 24 hours, use of aspirin in the past seven days, and elevated serum cardiac biomarkers. For the purpose of diagnostic accuracy analysis, a TIMI risk score > 4 was considered predictive of TVD based on prior literature applying this threshold for angiographic outcomes [9].

Coronary angiography was performed within 24-48 hours of admission in all patients unless contraindicated, in accordance with the institutional protocol for high-risk NSTEMI management. The procedure and interpretation of angiograms were performed by an experienced interventional cardiologist who was blinded to the TIMI risk score results. The primary angiographic outcome was the presence of TVD. TVD was strictly defined as the presence of ≥70% luminal diameter stenosis in all three major epicardial coronary arteries (the left anterior descending, left circumflex, and right coronary artery). This diagnostic criterion aligns with the standard anatomical definition used in contemporary guidelines and clinical trials [4].

Statistical analysis was performed using SPSS version 25.0 (IBM Corp., Armonk, NY). The diagnostic performance of the TIMI risk score (using the >4 cutoff) against the angiographic reference standard was evaluated by calculating sensitivity, specificity, positive predictive value (PPV), negative predictive value (NPV), and overall accuracy with 95% confidence intervals (CIs) using 2×2 contingency tables. Exploratory post hoc analyses included independent samples t-tests to compare mean TIMI risk scores across demographic and clinical subgroups. A p-value of <0.05 was considered statistically significant.

## Results

A total of 185 patients with NSTEMI were included, with a mean age of 62.78±9.29 years. Baseline characteristics are summarized in Table 1. There were 130 (70.3%) male patients and 55 (29.7%) female patients in this cohort. Hypertension was recorded in 86 (46.5%) patients, diabetes in 155 (83.8%), and a history of smoking in 75 (40.5%). Overall, 125 (67.6%) were urban residents. The mean BMI and LDL cholesterol were 26.4±3.8 kg/m² and 132.5±38.7 mg/dL, respectively.

**Table 1:** Baseline characteristics of the study participants (N=185) Data are presented as numbers (%) or means±SD. BMI: body mass index, LDL: low-density lipoprotein, MI: myocardial infarction, SD: standard deviation

Characteristic	Category	Number (%)/mean±SD
Age (years)		62.78±9.29
Gender	Male	130 (70.3%)
	Female	55 (29.7%)
Residential status	Urban	125 (67.6%)
	Rural	60 (32.4%)
BMI (kg/m²)		26.4±3.8
Hypertension		86 (46.5%)
Diabetes		155 (83.8%)
Smoking		75 (40.5%)
Family history of MI		42 (22.7%)
LDL cholesterol (mg/dL)		132.5±38.7

Coronary angiography demonstrated the presence of TVD in 158 (85.4%) patients. Patients with angiographically demonstrated TVD had a significantly higher mean TIMI risk score (5.8±1.2) compared to patients without TVD (3.1±1.4, p<0.001).

Applying a diagnostic threshold of TIMI risk score > 4, the cross-tabulation with angiography (Table 2) revealed that among 122 patients showing positive TIMI test results, 113 were true positives and nine were false positives. On the contrary, 45 of the 63 patients with a TIMI risk score ≤ 4 were false negatives and 18 true negatives. Using this distribution, the performance of the TIMI risk score for diagnosis was as follows: sensitivity, 71.5% (95% CI: 64.2-78.0); specificity, 66.7% (95% CI: 65.8-81.2), positive predictive value, 92.6% (95% CI: 87.9-96.1); negative predictive value, 28.6% (95% CI: 26.5-42.8); and overall accuracy, 70.8% (95% CI: 65.7-78.4).

**Table 2:** Cross-tabulation of TIMI score versus angiography for TVD Data are presented as numbers (%). TIMI: Thrombolysis in Myocardial Infarction, TVD: three vessel disease

TIMI score > 4	TVD on angiography		Total
	Yes (number (%))	No (number (%))	
Yes	113 (61.1%)	9 (4.9%)	122 (65.9%)
No	45 (24.3%)	18 (9.7%)	63 (34.1%)
Total	158 (85.4%)	27 (14.6%)	185 (100%)

Several significant associations were found in exploratory post hoc analyses. The mean TIMI risk scores stratified by demographic and clinical characteristics are reported in Table 3. The mean TIMI risk score for male patients was significantly higher than that of female patients (5.3±1.5 and 4.6±1.3, p=0.032). Diabetics also had higher scores than non-diabetics (5.4±1.4 versus 4.5±1.3, p=0.041). Urban dwellers had significantly higher scores than their rural counterparts (5.6±1.4 versus 4.8±1.2, p=0.028). No significant differences were seen based on hypertension status, smoking history, and family history of MI.

**Table 3:** Stratification of mean TIMI scores by demographic and clinical variables Data are presented as mean±SD. P-values and test statistics (t-value for independent t-tests) are shown. A p-value < 0.05 was considered significant. MI: myocardial infarction, SD: standard deviation, TIMI: Thrombolysis in Myocardial Infarction

Variable	Category	Mean TIMI score±SD	Test statistic (t)	p-value
Gender	Male	5.3±1.5	2.17	0.032
	Female	4.6±1.3		
Diabetes	Yes	5.4±1.4	2.06	0.041
	No	4.5±1.3		
Residence	Urban	5.6±1.4	2.22	0.028
	Rural	4.8±1.2		
Hypertension	Yes	5.2±1.5	1.25	0.215
	No	4.9±1.4		
Smoking	Yes	5.3±1.6	1.33	0.187
	No	5.0±1.3		
Family history of MI	Yes	5.1±1.4	0.75	0.456
	No	5.0±1.5		

## Discussion

This cohort elaborated the diagnostic value of the TIMI risk score in predicting TVD among patients suffering NSTEMI. A detailed post hoc analysis was performed to explore the determinants of TIMI risk score performance when applied in this setting. The results demonstrated moderate sensitivity (71.5%) and specificity (66.7%) for the TIMI risk score when using a cutoff value greater than 4. It is noteworthy that our score demonstrated a high positive predictive value (PPV) of 92.6%, denoting that patients with a score above this value were very likely to suffer from TVD. The negative predictive value (NPV) is, by contrast, much lower (28.6%), indicating that more than one-third of patients with a TIMI risk score ≤ 4 still had angiographically confirmed TVD.

These results are comparable to other findings of previous studies on the performance of the TIMI risk score in a comparable population. The results highlight the complex relationship between clinical risk scoring systems and the anatomical severity of coronary artery disease, emphasizing the importance of integrating clinical judgment with risk score interpretation [8,9,11]. The high PPV found indicates that in a high-prevalence scenario like ours, TIMI risk score > 4 likely represents TVD and could possibly guide early decisions for invasive strategy. The NPV of 34.2%, however, reveals a major clinical limitation, with more than two-thirds of subjects with TIMI risk scores ≤ 4 still having TVD. This result emphasizes that the TIMI risk score could not consistently rule out obstructive coronary disease and cannot be used to refuse cardiac angiography when clinically indicated.

While the identification of a high likelihood of triple vessel disease could theoretically reinforce the indication for early invasive strategy, it is important to acknowledge that current NSTEMI guidelines already recommend urgent angiography for high-risk patients based on established criteria (e.g., dynamic ECG changes, hemodynamic instability, refractory angina, or a high GRACE/TIMI risk score for adverse events) rather than solely for predicted anatomical severity. Therefore, in many health systems, the clinical decision to proceed to angiography may not substantially change even when a high TIMI risk score strongly suggests TVD. However, in settings with limited catheterization laboratory access, this could help prioritize patients for earlier intervention, especially when other high-risk features are equivocal. Conversely, the low NPV underscores that a low TIMI risk score should not delay angiography when clinically indicated, as it cannot reliably exclude extensive disease.

There were several noteworthy associations observed in our exploratory post hoc analyses. The significantly higher TIMI risk scores in male patients are consistent with published data, which demonstrated higher TIMI risk scores and more extensive coronary disease involvement in the male population [12]. The correlation with diabetes is likely due to the accelerated development of atherosclerosis and more extensive patterns of coronary disease that are observed in diabetic patients [13]. The rural-urban difference in TIMI risk scores might result from inequalities of lifestyle, access to healthcare, and distribution of risk factors that require exploration in the Pakistani population [14].

Our findings suggest that the TIMI risk score alone should not determine the need for angiography, given its low NPV (34.2%), which fails to exclude TVD in over one-third of patients with a score ≤4. However, its high PPV (92.8%) may still offer clinical utility by reinforcing the indication for earlier invasive evaluation in patients with a score >4, particularly in high-prevalence settings where resource prioritization is required. The TIMI risk score was originally designed to predict adverse clinical outcomes rather than angiographic severity [5]. Therefore, the TIMI risk score ought to complement, not supplant, clinical judgment and guideline-based decisions on angiography. Future investigations could explore combined models that merge clinical scoring systems with other non-invasive indicators for more refined patient stratification before invasive evaluation.

Several limitations merit acknowledgment. The single-center nature of the study and the small number of cases may restrict generalizability. The high prevalence of TVD (85.4%) in our study cohort, although representative of the tertiary care patient population, could serve to exaggerate PPV and underestimate NPV. The TIMI risk score cutoff of >4 was derived from outside literature, and local validation of the best-defined threshold is required. Further, the cross-sectional study does not allow us to reflect on long-term effects.

## Conclusions

The TIMI risk score has fair diagnostic accuracy in predicting triple vessel disease among patients with NSTEMI, with high positive predictive value but low negative predictive value in a setting of high pretest probability. It could be of value as an initial screening and, more importantly, to detect high-risk patients who would need urgent intervention. However, its inability to reliably exclude extensive coronary disease necessitates that clinical judgment and timely angiography remain central to patient care. Future multicenter studies with larger cohorts are recommended to validate population-specific thresholds and develop integrated predictive models.
